# Chronic Intermittent Ethanol Regulates Hippocampal GABA(A) Receptor Delta Subunit Gene Expression

**DOI:** 10.3389/fncel.2015.00445

**Published:** 2015-11-09

**Authors:** Paolo Follesa, Gabriele Floris, Gino P. Asuni, Antonio Ibba, Maria G. Tocco, Luca Zicca, Beniamina Mercante, Franca Deriu, Giorgio Gorini

**Affiliations:** ^1^Department of Life and Environmental Sciences, University of CagliariCagliari, Italy; ^2^Department of Public Health, Clinical and Molecular Medicine, University of CagliariCagliari, Italy; ^3^Department of Biomedical Sciences, University of SassariSassari, Italy

**Keywords:** alcoholism, dependence, addiction, gene expression, GABA

## Abstract

Chronic ethanol consumption causes structural and functional reorganization in the hippocampus and induces alterations in the gene expression of gamma-aminobutyric acid type A receptors (GABA_A_Rs). Distinct forced intermittent exposure models have been used previously to investigate changes in GABA_A_R expression, with contrasting results. Here, we used repeated cycles of a Chronic Intermittent Ethanol paradigm to examine the relationship between voluntary, dependence-associated ethanol consumption, and GABA_A_R gene expression in mouse hippocampus. Adult male C57BL/6J mice were exposed to four 16-h ethanol vapor (or air) cycles in inhalation chambers alternated with limited-access two-bottle choice between ethanol (15%) and water consumption. The mice exposed to ethanol vapor showed significant increases in ethanol consumption compared to their air-matched controls. GABA_A_R alpha4 and delta subunit gene expression were measured by qRT-PCR at different stages. There were significant changes in GABA_A_R delta subunit transcript levels at different time points in ethanol-vapor exposed mice, while the alpha4 subunit levels remained unchanged. Correlated concurrent blood ethanol concentrations suggested that GABA_A_R delta subunit mRNA levels fluctuate depending on ethanol intoxication, dependence, and withdrawal state. Using a vapor-based Chronic Intermittent Ethanol procedure with combined two-bottle choice consumption, we corroborated previous evidences showing that discontinuous ethanol exposure affects GABA_A_R delta subunit expression but we did not observe changes in alpha4 subunit. These findings indicate that hippocampal GABA_A_R delta subunit expression changes transiently over the course of a Chronic Intermittent Ethanol paradigm associated with voluntary intake, in response to ethanol-mediated disturbance of GABAergic neurotransmission.

## Introduction

Prolonged excessive ethanol (EtOH) consumption can lead to increased risk of dependence. Critical neurobiological changes have been suggested to contribute to the transition from EtOH consumption and abuse to dependence, leading to behavioral abnormalities, which ultimately promote and sustain high levels of compulsive drinking (Vengeliene et al., 2009; Heilig et al., 2010; Koob and Volkow, 2010; Gorini et al., 2014; Krishnan et al., 2014). Moreover, EtOH self-administration behavior can be influenced by perturbations of the neuroendocrine pathways related to reward, stress, and anxiety (Koob, 2003).

The hippocampus, important for cognitive function, stress, and emotional regulation (Bannerman et al., 2014), has been associated with seeking of and reward from EtOH (Koob and Volkow, 2010), with evidence of structural and functional reorganization following chronic EtOH exposure. Multiple neurotransmitter systems are affected during the development of EtOH dependence, including the GABAergic system. A considerable body of evidence suggests that GABA_A_Rs mediate a few of the acute and chronic actions of EtOH (Faingold et al., 1998; Ueno et al., 2001; Harris et al., 2008; Trudell et al., 2014), and neuroactive steroids have been shown to be implicated in these actions (Morrow et al., 2001), even in isolated hippocampal slices (Sanna et al., 2004). Given that EtOH can target GABA_A_Rs and modulate their function, the subunit compositions of receptor subtypes play a crucial role in responses to neurobiological effects of EtOH in specific brain areas, neuronal populations, and synaptic localizations. GABA_A_R gene expression is influenced by physiological processes, environmental stimuli, and drugs (Fénelon and Herbison, 1996; Holt et al., 1996; Concas et al., 1998; Smith et al., 1998b; Sanna et al., 2003; Follesa et al., 2004; Biggio et al., 2009; Whissell et al., 2015), and chronic EtOH exposure and withdrawal induce a shift in excitatory/inhibitory tone, with decreased GABAergic inhibition (Kumar et al., 2009). Distinct GABA_A_R subtypes participate in the phasic and tonic inhibitory control of neuronal activity, and those associated with phasic inhibition are located at the synapses and are modulated by higher EtOH doses. In contrast, the subtypes configured with α4/α6 and δ subunits are located exclusively at extrasynaptic sites, where their continuous activation elicits tonic GABAergic inhibition (Farrant and Nusser, 2005; Glykys et al., 2008; Brickley and Mody, 2012). Such extrasynaptic GABA_A_R subtypes (and thus α4 and δ subunits) are expressed preferentially in the dentate gyrus of the hippocampus, thalamus, and cerebellar granule cells (Semyanov et al., 2004), with lower levels in the cortex, striatum, CA1 region, and other brain areas (Pirker et al., 2000). Their distinctive properties include a higher affinity for GABA (Saxena and Macdonald, 1996; Brown et al., 2002), higher sensitivity to neuroactive steroids (Adkins et al., 2001; Brown et al., 2002; Wohlfarth et al., 2002; Mody, 2008; Sarkar et al., 2011), and controversially, to acute EtOH (Sundstrom-Poromaa et al., 2002; Wallner et al., 2003) when compared to other GABA_A_R subtypes.

The involvement of the hippocampal GABAergic system in alcoholism has been emphasized by comparing differential GABAergic gene expression within the hippocampus of alcoholics and in a rat model of vulnerability to EtOH dependence (Enoch et al., 2012), with congruent findings. Furthermore, several studies have shown that hippocampal α4 and δ subunit levels are affected by excessive EtOH consumption and subsequent withdrawal. In rat hippocampus, α4 peptide levels were upregulated in a region- and time-dependent manner 24 h after a voluntary 40-day EtOH-based liquid diet (Matthews et al., 1998). Similarly, a 48-h withdrawal period after protracted (60 days) forced EtOH consumption led to a significant increase in α4 subunit mRNA levels in the dentate gyrus, CA3, and CA1 regions (Mahmoudi et al., 1997), accompanied by a general increase in α4 and γ2 peptide levels and decreases in α1 and δ peptide levels (Cagetti et al., 2003). Moreover, there has been evidence of significant increases in the α1, α4, α5, β1, and γ1 subunit mRNAs in the post-mortem hippocampal dentate gyrus region of individuals suffering from long-term alcohol dependence (≥80 g alcohol per day; Jin et al., 2012). Notably, hippocampal overexpression of the α4 subunit has been associated with increased anxiety (Smith et al., 1998a), which is thought to contribute to increased vulnerability to EtOH abuse. All these changes might alter the GABAergic tone and induce a state of hyperexcitability (Kumar et al., 2009; Olsen and Spigelman, 2012).

However, other studies have indicated dissimilar results in both rodent and human hippocampi. Diverse forced intermittent exposure paradigms had no effects on hippocampal α4 or δ subunit gene expression during rat adulthood (Centanni et al., 2014; Van Skike et al., 2015). Furthermore, α4 transcript levels were not significantly altered in the hippocampal body of alcoholics or in the total hippocampus of rats genetically predisposed to EtOH dependence (Enoch et al., 2012).

Overall, these studies provide evidence that excessive EtOH exposure can have multifaceted effects on hippocampal GABA_A_R subunit expression. Nevertheless, differences in intermittent exposure/withdrawal durations and consumption methods (intragastric intubation, injection, liquid diet as sole source of calories and fluid) lead to distinct patterns of molecular changes in specific subregions, which do not necessarily account for vulnerability to EtOH in rodents or contribute to dependence in alcoholics. In the last decade, better procedures for modeling alcohol dependence and relapse drinking have been developed by combining passive EtOH vapor exposure with subsequent voluntary, free-choice consumption (Becker and Ron, 2014). In addition, it has been found that the use of inbred mouse strains results in higher EtOH consumption and blood EtOH concentrations compared to other rodent models.

Previously, we have shown that both chronic EtOH treatment in cultured neurons (Follesa et al., 2005) and voluntary two-bottle choice (2BC) EtOH consumption in mice (Sanna et al., 2011) increase the abundance of hippocampal GABA_A_R δ subunit mRNA and protein. Here, we used repeated cycles of a Chronic Intermittent EtOH (CIE) exposure in combination with a 2BC paradigm to examine the relationships between voluntary, dependence-associated EtOH consumption, its resulting blood EtOH levels, and GABA_A_R α4 and δ subunit expressions in mouse hippocampus. In this paradigm, forced EtOH vapor exposure is employed to achieve and maintain high blood EtOH concentrations (BECs) in C57BL/6J mice, and such exposure results in increased self-administration of EtOH (Becker and Lopez, 2004). Four repeated bouts of EtOH exposure and abstinence were used because they result in more permanent changes in the reinforcing effects of EtOH (Roberts et al., 2000; Lopez and Becker, 2005). Previous studies using this model have suggested that the development of alcohol dependence and withdrawal involves brain region-specific and time-dependent profound neuroadaptive alterations in protein (Gorini et al., 2013; Uys et al., 2015) and gene (Melendez et al., 2012; Osterndorff-Kahanek et al., 2015) expression, and these alterations seem to be more pronounced 8 h after EtOH exposure. Hence, we measured transcript levels at different time points in the paradigm to better understand how drinking behavior could affect GABA_A_R expression. To our knowledge, no study has yet measured hippocampal GABA_A_R expression at different time points during and after voluntary EtOH consumption following forced EtOH vapor exposure in mice by comparing molecular data with concomitant BECs.

## Materials and Methods

### Subjects

Adult male C57BL/6J mice were bred in our animal facility under an artificial 12:12 light/dark cycle at a constant temperature of 23°C, 65% humidity, and *ad libitum* availability of rodent food and water. After birth in our animal facility, mouse pups were left undisturbed with their mothers until weaning (21 days after birth). At the age of 13 weeks, male mice were selected and housed in a dedicated room with a different 12:12 reverse light/dark cycle (off at 10:30 h). In the 14th week, the mice were used to perform all the procedures listed below. Body weight was recorded daily while the mice were consuming EtOH and during CIE exposure cycles. The mice were housed individually when measuring fluid consumption and regrouped at the end of each session, and they were not deprived of food or water at any time during the experiment. This study was carried out in accordance with the recommendations of the “Guidelines for care and use of experimental animals” issued by the Italian Ministry of Health (D.L. 26/2014), by the European Union (2010/63/UE) and the “Guide for Care and Use of Laboratory Animals”, adopted by the NIH, USA (8th edition, 2011). The protocol was approved by the “Committee on Animal Use and Care” of the University of Cagliari.

### General Paradigm Design

**FIGURE 1:**
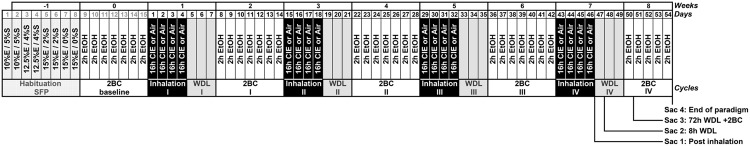
**Simplified schematic representation of Chronic Intermittent Ethanol (CIE) paradigm combined with voluntary 2BC ethanol (EtOH) intake.** Following habituation [with sucrose-fading procedure (SFP)] and baseline periods, the mice were maintained in inhalation chambers for 4 days and exposed to EtOH vapor intermittently (CIE group, 4 × 16 h EtOH + 8 h Air) or constant air (Air group). After 72-h of abstinence (WDL) following each inhalation cycle, all mice were tested for 2 h/d 2BC limited-access EtOH consumption for 7 days. Each 4-day CIE/air exposure cycle was followed by a 7-day limited-access 2BC drinking test, and this sequence was repeated four times. The last 2BC drinking test lasted 5 days only, leading to a total of 54 days after the end of the baseline period. Samples were collected twice on day 47 (Sac 1 at 09:30 h, “Post inhalation”, and Sac 2 at 17:30 h, “8 h WDL”), and then on days 50 (Sac 3 at 12:00 h, “72 h WDL + 2BC”) and 54 (Sac 4 at 12:00 h, “End of paradigm”), as indicated in the section “Materials and Methods.”

The general study design involved the use of an EtOH dependence and relapse drinking model developed by Dr. Becker’s laboratory (Becker and Lopez, 2004; Lopez and Becker, 2005; Griffin et al., 2009a,b). Briefly, the mice were randomly divided in two groups, and both groups were first trained to drink EtOH using a 2BC limited-access procedure (described below) to stabilize their daily EtOH intake. After this baseline period, the groups were maintained in inhalation chambers for 4 days and exposed to EtOH vapor (CIE group) or air. After a 72-h abstinence period following each inhalation cycle, the CIE and the air-matched control mice (Air) were tested for 2 h/d 2BC limited-access EtOH consumption for 7 days. In short, each 4-day CIE/air exposure cycle was followed by a 7-day limited access drinking test, and this sequence was repeated four times (**Figure 1**). Five separate experiments involving a total of EtOH (*n* = 63) and Air (*n* = 40) mice were conducted for testing voluntary ethanol consumption over four test cycles of CIE exposure.

### Habituation

Using a modified sucrose-fading procedure (SFP; Samson, 1986), the mice were given daily access to EtOH for 2 h in the home cage 0.5 h prior to the start of the dark cycle to stabilize their daily intake. During the 2-h limited access period, the mice were presented with a 2BC to drink EtOH or tap water, and the positions of the bottles were alternated on a daily basis. Every day during the 2-h procedure, the single standard water bottle was removed from each cage and replaced with two 250 ml bottles, one containing EtOH/sucrose solutions and the other containing water/sucrose at matching sucrose concentration during the first 6-day period of the SFP and just water or EtOH thereafter as follows: 10% EtOH/5% sucrose for 2 days, 12.5% EtOH/4% sucrose for 2 days, 15% EtOH/2% sucrose for 2 days, and 15% EtOH/0% sucrose as a final solution for 9 days. At the end of each 2-h access period, the EtOH bottles and the water bottles were removed, and the one standard water bottle was returned to the home cage. The EtOH (v/v) solutions were prepared by mixing 95% EtOH with deionized water and adding sucrose (w/v) when appropriate. All solutions were prepared daily and presented at room temperature. EtOH intake was measured daily by weighing the bottles. Differences with the initial weight were calculated to establish the amount of EtOH consumed during the 2-h period.

### CIE Exposure

Chronic Intermittent EtOH exposure was obtained by using inhalation chambers as described previously (Becker and Lopez, 2004; Lopez et al., 2012). Briefly, ethanol (95%) was volatilized, mixed with fresh air, and delivered to the chambers at a rate of 10 L/min, resulting in vapor concentrations of 15–20 mg/L. These inhalation conditions have been shown to yield stable BECs (150–200 mg/dl) during each cycle of intoxication in this mouse strain (Griffin et al., 2009a). The CIE mice were exposed to a 4 -day cycle of EtOH vapor for 16 h/d (from 17:30 to 09:30 h on the following day, no exposure for the remaining 8 h). The Air mice were subjected to the same chamber conditions with air instead of EtOH vapor. The housing conditions in the inhalation chambers were identical to those in the colony room. To maintain a high, stable level of intoxication during each cycle of EtOH vapor exposure (Griffin et al., 2009a), the CIE group received injections of a loading dose of EtOH (1.6 g/kg) and the alcohol dehydrogenase inhibitor pyrazole (1 mmol/kg, intraperitoneally in a volume of 0.02 ml/g body weight) before placement into the EtOH vapor chambers. Similarly, the mice in the Air group were administered saline and pyrazole before being placed into the control chambers. All mice received daily pyrazole injections before their final removal from the chambers.

### 2BC Limited-access Drinking

After 72 h of abstinence following each inhalation cycle, all mice were tested for 2 h/d, 2BC limited-access EtOH consumption for seven consecutive days. As during the SFP, the 2-h daily drinking sessions included 30 min of light and 1.5-h of darkness (from 10:00 to 12:00 h). The mice were housed individually for 2 h with access to two drinking bottles, one containing 15% v/v EtOH and the other containing tap water. The positions of the bottles was switched daily, and the amount of EtOH consumed by each mouse was recorded and converted to g/kg based on the grams of EtOH consumed and body weight. Following the 2 h of 2BC testing, the mice were regrouped. The dependent variables recorded and analyzed include EtOH intake (gram and gram per kilogram). Water intake and total fluid intake were measured as well (data not shown).

### Tissue Collection

Samples were collected (in the dark when needed) at four different time points: immediately after the end of the last EtOH vapor exposure (day 47, 09:30 h; referred as “Post inhalation”) in the fourth cycle, 8 h after the end of the last EtOH vapor exposure (day 47, 17:30 h; “8 h WDL”), at the end of the first subsequent 2BC drinking session (day 50, 12:00 h; “72 h WDL + 2BC”), and at the end of the last 2BC drinking session (day 54, 12:00 h, “End of paradigm”).

Blood and brain tissues were sampled to measure EtOH concentrations and GABA_A_R subunit mRNA levels. Bilateral hippocampi were dissected, frozen in dry ice, and stored at –80°C until they were used for assays, as described below. An amount of samples sufficient to perform a powerful statistical analysis were collected from a limited number of mice belonging to different experimental groups (as indicated in the figure legends).

### Blood Ethanol Assays

Immediately after rapid decapitation, blood samples were collected from the encephalic trunk using two 70 μL heparinized capillary tubes per animal, transferred to 1.5 mL tubes containing 10 μL heparin, and mixed for BEC analysis. One hundred microliters of aliquots in vials were then subjected to gas chromatography, as described previously (De Martinis and Martin, 2002; Maeda et al., 2006). Sample concentrations were determined by interpolating a standard curve; this method detects concentrations of up to 500 μM EtOH. Briefly, standards were prepared by diluting ethanol in ACSF and 0.75 M perchloric acid to mimic sample harvesting. Before the assay, 2 μL of the obtained standards were aliquoted in vials and heated at 65°C for 45 min with the samples. Then, 1 mL aliquot from each vial was injected into the gas chromatography column, and peak heights were recorded on a chromatogram. Blood ethanol levels were expressed in units of mg/dl.

### Measurement of GABA_A_R Subunit mRNA Levels by qRT-PCR

Total RNA was extracted from the frozen hippocampi by using the guanidine isothiocyanate method (Chomczynski and Sacchi, 2006) and was quantified by measuring absorbance at 260 nm. The yield and quality of the isolated RNA was determined by agarose gel electrophoresis. Single-stranded cDNA was synthesized from total RNA using the *iScript^TM^ cDNA Synthesis* kit (Bio-Rad, Hercules, CA, USA) according to the manufacturer’s instructions. The synthesized cDNA was diluted 10 times and used to determine GABA_A_R subunit mRNA levels. Following reverse transcription, quantitative RT-PCR (qRT-PCR) was performed in triplicate using the *iQ SYBR Green Supermix* (Bio-Rad) PCR mix containing 100 mM KCl, 40 mM Tris-HCl pH 8.4, 0.4 mM of each dNTP, 50 U/mL DNA Polymerase (iTaq), 6 mM MgCl_2_, SYBR Green I, 20 nM fluorescein, and stabilizers. The reaction was performed using a thermocycler (Real-Time PCR detection system C1000/CFX96, Bio-Rad, Hercules, CA, USA) in a final volume of 25 μL (5 μL RNase-free H_2_O, 5 μL cDNA template, 2.5 μL of each primer, and 12.5 μL of 2x *iQ SYBR^TM^ Green Supermix*) under the following PCR conditions: initial heating at 95°C for 3 min to denature the cDNA and activate the *Taq* DNA Polymerase, followed by 40 cycles consisting of denaturation at 95°C for 30 s, annealing at 60°C for 60 s, and extension at 72°C for 2 min. The reaction was then stopped with a final step at 72°C for 15 min. Qiagen QuantiTect Primer Assay systems with the following 10X primers were used: GABA_A_R α4 (product number 249900, NM_010251, final conc. 1X), and GABA_A_R δ (product number 249900, NM_008072, final conc. 1X). Beta-actin (product number 249900, NM_007393, final conc. 1X), and GAPDH (product number 249900, NM_008084, final conc. 1X) were used as endogenous control genes. The analysis was performed using the CFX Manager software (Bio-Rad) for calculating gene expression in the analysis mode ΔΔC(t) according to the manufacturer’s instructions.

### Data and Statistical Analysis

The data are presented as mean ± SEM and compared by one-way or two-way analysis of variance (ANOVA) and an appropriate test, as indicated in figure legends, by using Prism software (version 6, GraphPad) or STATISTICA (version 7, StatSoft Inc.).

Measures of ethanol intake (g/kg/2 h) were averaged over the 7-day limited access sessions during baseline and each of the test cycles for each subject. These data were analyzed by two-way ANOVA, with Group (CIE vs. Air) as a between-subjects factor and Test Cycle as a repeated measure. For *post hoc* multiple comparisons, we used the Newman–Keuls, Bonferroni, or Dunnett tests, as appropriate, or the *t*-test and the *F* test to compare variances in single-group comparisons, as indicated in figure legends.

Ethanol concentration in blood samples and GABA_A_R gene expression were also analyzed using Student’s *t*-tests, one or two-way ANOVA (as appropriate and indicated in figure legends), followed by the Bonferroni test. Correlation analyses were conducted using Pearson’s Product Moment analysis. For all analyses, significance levels were set at *p* < 0.05.

## Results

**FIGURE 2:**
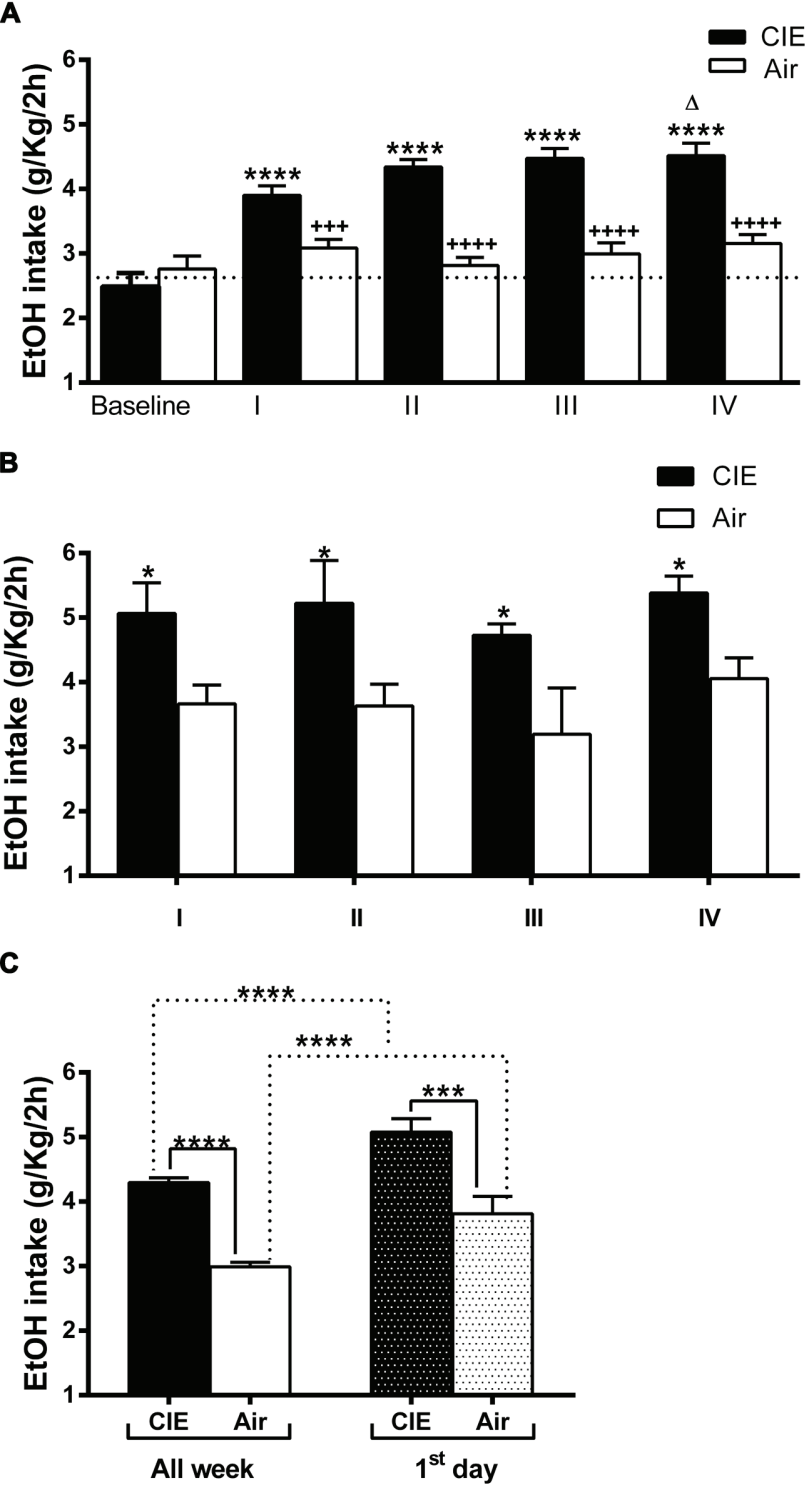
**Effect of repeated cycles of CIE exposure on voluntary 2BC EtOH intake over time.** Forced exposure to EtOH vapor increased average EtOH consumption in the CIE mice compared to the Air controls. The average EtOH intake is expressed in g/kg/2 h. The values are mean ± SEM. **(A)** Voluntary EtOH consumption was compared between the CIE (full bars) and the Air (empty bars) mice during baseline and each of the four 2BC weekly cycles (I, II, III, and IV). Repeated two-way ANOVA measurements revealed a significant treatment effect [CIE (*n* = 63) versus Air (*n* = 40), *F*(1,101) = 48.060, *p* = 0.000001], significant cycle effect [*F*(4,404) = 15.608, *p* = 0.000001], and significant treatment × test cycle interaction [*F*(4,404) = 3.676, *p* = 0.00591]. The *post hoc* Newman–Keuls test revealed significant differences (^∗∗∗∗^*p* < 0.0001) between each cycle and the baseline in the CIE but not in the Air mice, whose average consumption was steady over time. The Bonferroni multiple comparison test showed significant differences in the amount of EtOH consumed between the Air and the CIE mice in the corresponding cycle (^+++^*p* < 0.001 and ^++++^*p* < 0.0001) and a difference within the CIE group (Δ*p* < 0.05) in the amount of EtOH consumed between cycles I and IV. **(B)** Voluntary EtOH intake during the first day of each 2BC session in the CIE (full bars) and the Air (empty bars) mice. Two-way ANOVA revealed a significant treatment effect [CIE (*n* = 41) versus Air (*n* = 35), *F*(1,68) = 22.11, *p* = 0.0001], non-significant cycle effect [*F*(3,68) = 1.001, *p* = 0.3977], and non-significant treatment × test cycle interaction [*F*(3,68) = 0.03780, *p* = 0.9901]. The Bonferroni multiple comparison test showed significant differences (^∗^*p* < 0.05) between the Air and the CIE mice in the corresponding cycle. **(C)** Comparison of average amount of globally consumed EtOH throughout the week (All week, full and empty bars) and on each first day (first day, dark and light gray bars) across all four 2BC tests in the two groups. Two-way ANOVA revealed a significant treatment effect [*F*(1,175) = 1010, *p* < 0.0001], significant first day/all week effect [*F*(1,175) = 2593, *p* < 0.0001], and non-significant interaction [*F*(1,175) = 0.5996, *p* = 0.4398]. The Bonferroni multiple comparison test showed significant differences (^∗∗∗∗^*p* < 0.0001) between the compared groups. Further, analysis revealed significant effects of the CIE treatments on both all week [(^∗∗∗∗^*p* < 0.0001); *t*-test: *t* = 12.04 (101); *R*^2^ = 0.5895 and *F* test to compare variances *F* = 1.713 (62,39)] and first day [(^∗∗∗^*p* < 0.001); *t*-test: *t* = 3.761 (74); *R*^2^ = 0.1606 and *F* test to compare variances *F* = 1.416 (40,34)].

To determine the effect of forced exposure to EtOH vapor on 2BC voluntary intake, we monitored daily EtOH consumption (expressed in g/kg/2 h). Daily intake values were averaged over the 7 days of each 2BC drinking test period. The data obtained were analyzed to evaluate the 2BC drinking of the CIE vs. the Air group after each of the four forced exposure cycles. In agreement with previous findings (Becker and Lopez, 2004; Lopez and Becker, 2005), repeated cycles of CIE exposure increased voluntary EtOH intake in general. Although the baseline intake was similar for both groups (2.49 ± 0.21 CIE; 2.76 ± 0.19 Air; average 2.63 ± 0.14 g/kg) and drinking in the Air mice remained relatively unchanged, the average EtOH consumption was higher in mice subjected to forced EtOH vapor exposure, reaching ∼4.5 g/kg in the IV cycle (**Figure 2A**). This was supported by repeated ANOVA measurements, which revealed a significant treatment effect (CIE versus Air) [*F*(1,101) = 48.060, *p* = 0.000001], significant cycle effect [*F*(4,404) = 15.608, *p* = 0.000001], and significant treatment × test cycle interaction [*F*(4,404) = 3.676, *p* = 0.00591]. The *post hoc* Newman–Keuls test revealed that following the first vapor/control chamber exposure, 2BC EtOH consumption in the EtOH vapor-exposed mice (CIE) increased significantly (+26 ± 4.1%, *p* < 0.0001) relative to the control mice (Air) (**Figure 2A**). This effect was greater after the second chamber exposure, with *post hoc* analysis (*p* < 0.0001) revealing increased EtOH consumption (+54 ± 4.2%) in CIE mice relative to Air mice across the second 7-day 2BC limited-access drinking period. Similar significant increases were evident even during the third (+49 ± 5%, *p* < 0.0001) and fourth (+43 ± 6.3%, *p* < 0.0001) 2BC test periods. In addition, the *post hoc* analysis revealed a significant increase in EtOH consumption between cycles I and IV (*p* < 0.05).

Daily EtOH intake across test periods was also compared to understand behavioral effects related specifically to the 72-h withdrawal period following inhalation and before the 2BC sessions. The average EtOH consumption during the first day of each 2BC test period was always higher in CIE group compared to the Air controls (*p* < 0.05, **Figure 2B**). This was supported by the results of repeated ANOVA measurements, which revealed a significant treatment effect (CIE versus Air) [*F*(1,68) = 22.11, *p* = 0.0001], non-significant cycle effect [*F*(3,68) = 1.001, *p* = 0.3977], and non-significant treatment × test cycle interaction [*F*(3,68) = 0.03780, *p* = 0.9901].

The total amount of EtOH consumed across all four tests was significantly higher in the CIE mice (+44 ± 2.5%, *p* < 0.0001; **Figure 2C**). The average daily EtOH consumption was 4.29 ± 0.07 g/kg/2 h in the CIE mice and 2.99 ± 0.07 g/kg/2 h in the Air mice. Moreover, the total amount of EtOH consumed on the first day across all four tests was significantly higher in the CIE mice (+33.2 ± 5.49%, *p* < 0.001) compared to that in the Air mice (**Figure 2C**). Furthermore, when comparing only the first-day global intake against the average weekly consumption, we found that both the CIE mice and the Air mice consumed greater amounts of EtOH during the first day (**Figure 2C**), with a significant treatment effect [*F*(1,175) = 1010, *p* < 0.0001], significant first day/all week effect [*F*(1,175) = 2593, *p* < 0.0001], and non-significant interaction [*F*(1,175) = 0.5996, *p* = 0.4398]. The overall EtOH consumption patterns were consistent with those observed in similar previous experiments, showing that repeated exposure to EtOH vapor is associated with subsequent increases in EtOH consumption under 2BC conditions.

**FIGURE 3:**
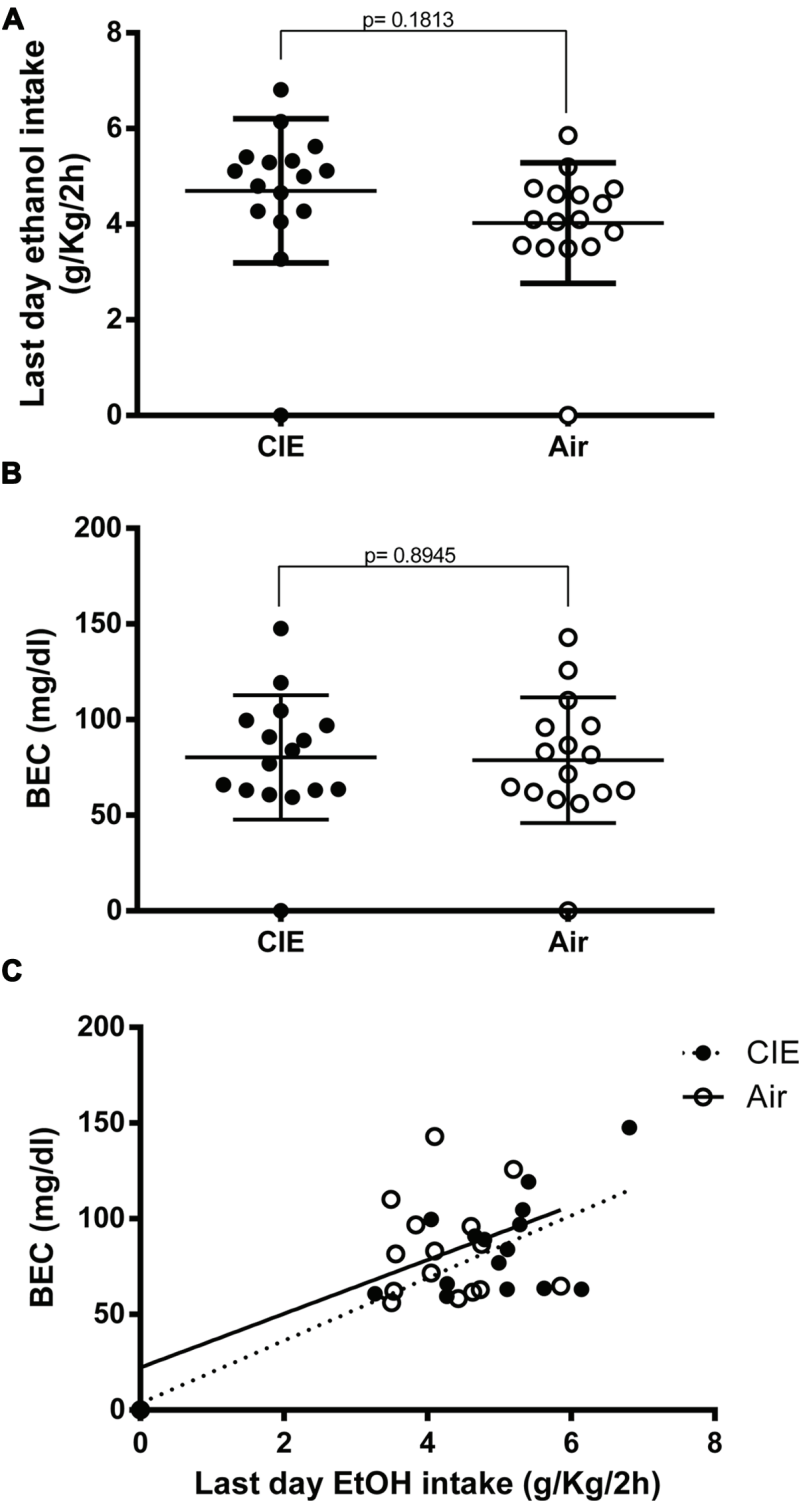
**Final effects of CIE exposure on voluntary EtOH intake and blood EtOH concentrations (BECs) at end of paradigm.** EtOH consumption was measured during the last day (day 54) immediately before sample collection. EtOH intake is expressed in g/kg/2 h, and BECs are expressed in mg/dl. The full dots represent the CIE samples, and the empty dots represent the Air samples. Values are mean ± SEM of 16 CIE and 16 Air mice. **(A)** On the last day of the paradigm, EtOH intake levels were similar in the two groups. A non-significant increase was still evident in the CIE mice compared to the Air mice [(4.696 and 4.024 g/kg/2 h, respectively); *p* = 0.1813; *t*-test: *t* = 1.368 (30); *R*^2^ = 0.05876 and *F* test to compare variances *F* = 1.422 (15,15)]. **(B)** Blood EtOH levels were measured immediately after sample collection (Sac 4). The differences between the average BECs of the two groups were not statistically different [*p* = 0.8945; *t*-test: *t* = 0.1337 (30); *R*^2^ = 0.0005955 and *F* test to compare variances *F* = 1.023 (15,15)]. **(C)** Correlation between the amount of EtOH intake by the CIE and the Air groups during the final session of the last 2BC test and the resultant BECs measured immediately after the drinking session. Pearson correlation analysis of the data showed *R*^2^ = 0.5736 for the CIE group and *R*^2^ = 0.2913 for the Air group (*p* = 0.0007 and *p* = 0.0309, respectively). Linear regression analysis showed a slope different from zero for the CIE group [*p* = 0.007; *F* = 18.83 (1,14)] and the Air group [*p* = 0.0309; *F* = 5.755 (1,14)]. The analysis also demonstrated no significant difference between slopes when comparing the two groups [*p* = 0.7398; *F* = 0.113352 (1,28)].

At the very end of our paradigm, immediately before sample collection (day 54, as denoted by the scheme in **Figure 1**), the CIE mice continued to show a small increase in EtOH intake, although this was not significant (*p* = 0.1813) compared to the controls (4.696 and 4.024 g/kg/2 h, respectively; **Figure 3A**). Blood ethanol levels were measured in samples obtained immediately after the final limited access session of the study (Sac 4 on day 54 in **Figure 1**); the average BECs of the two groups were similar (**Figure 3B**) and not statistically significant (*p* = 0.8945). Moreover, as shown in **Figure 3C**, the BECs were partially correlated to the average amount of EtOH consumed during the last-day 2-h access period (*R*^2^ = 0.5736; *p* < 0.001, and *R*^2^ = 0.2913; *p* < 0.05 for CIE and Air mice, respectively). Linear regression analysis showed a slope different from zero for the CIE [*p* = 0.007; *F* = 18.83 (1,14)] and the Air groups [*p* = 0.0309; *F* = 5.755 (1,14)]. The analysis also demonstrated no significant difference between slopes when comparing the CIE and the Air groups [*p* = 0.7398; *F* = 0.113352 (1,28)], indicating that repeated cycles of EtOH vapor exposure did not alter EtOH pharmacokinetics in the CIE mice. Collectively, these results suggest that when EtOH was presented in a 2BC limited-access paradigm, previous repeated, forced, chronic EtOH exposures and withdrawal experiences increased subsequent voluntary intake in the CIE compared to the Air mice, but this difference did not seem to last after prolonged access, at which point the groups showed similar EtOH intakes and blood levels.

**FIGURE 4:**
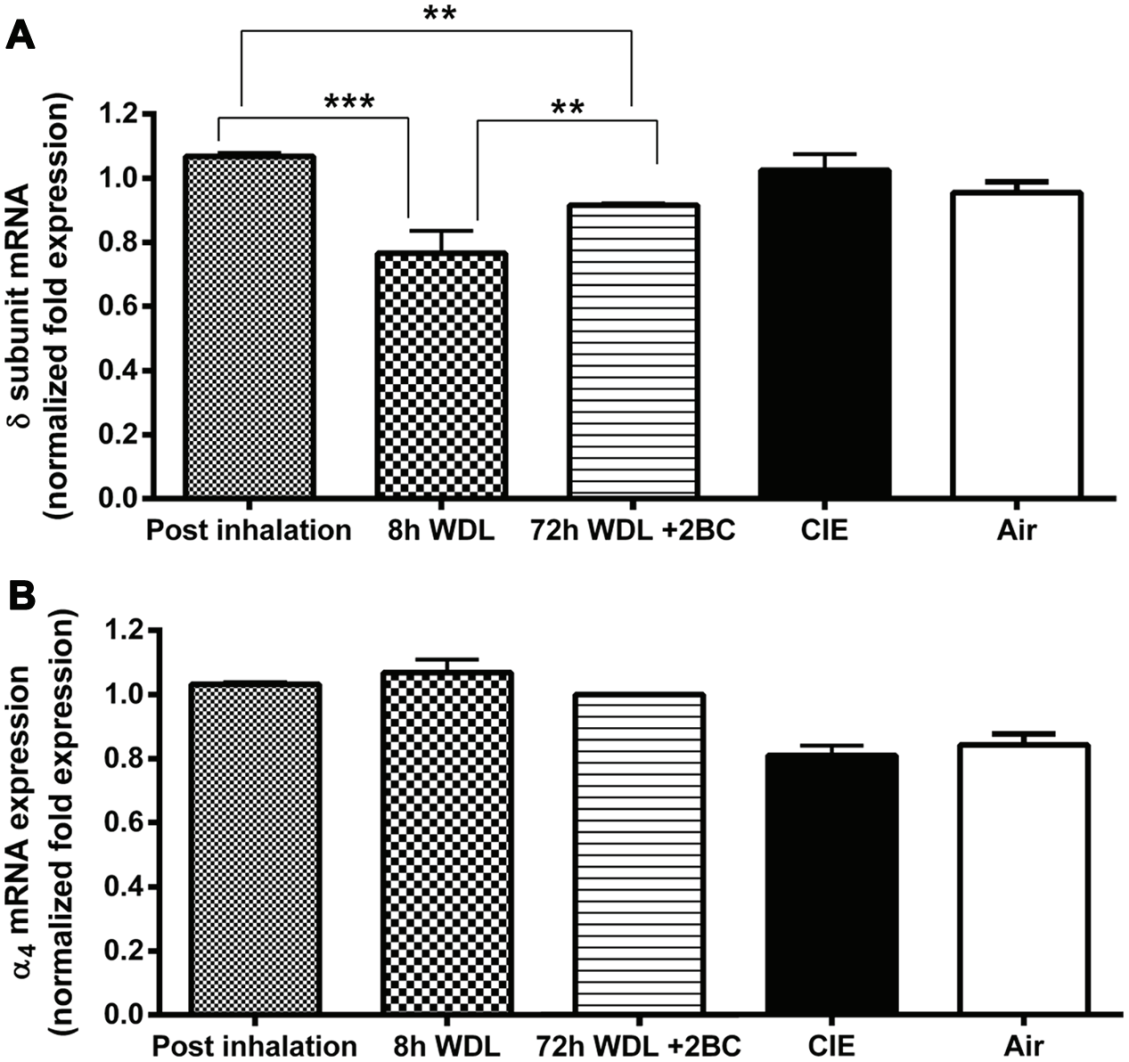
**Effects of repeated cycles of CIE paradigm on hippocampal GABA_A_R subunit mRNA expression at different time points.** Semi-quantitative data were obtained by qRT-PCR and gene fold expression levels were normalized against housekeeping mRNA. The first three bars in both graphs refer to the CIE mice immediately after the last forced exposure to EtOH vapor (Post inhalation), 8 h after the last exposure to EtOH vapor (8 h WDL), and right after the first subsequent 2BC drinking session (72 h WDL + 2BC). GABA_A_R mRNA expression levels are shown for the δ **(A)** and the α4 **(B)** subunits. Only the δ mRNA expression data showed statistical significance, as indicated. The last two bars in both graphs refer to fold expression in the CIE and the Air mice at the end of the paradigm, immediately after the last 2BC session in the last test week. At this stage, no statistical differences were observed between CIE (*n* = 10) and Air (*n* = 9) for both the δ [*p* = 0.2809; *t*-test: *t* = 1.114 (17); *R*^2^ = 0.06801 and *F* test to compare variances *F* = 2.458 (9,8)] and the α4 [*p* = 0.4750; *t*-test: *t* = 0.7305 (17); *R*^2^ = 0.03043 and *F* test to compare variances *F* = 1.076 (9,8)] subunit mRNA levels. Multiple comparison ANOVA of the first three time points showed significance between treatments with *p* < 0.0001, *F* = 46,41 (2,13), followed by the Bonferroni multiple comparison *post hoc* test, ^∗∗^*p* < 0.01; ^∗∗∗^*p* < 0.001. The values are means ± SEM in triplicates of 4, 8, and 4 mice for post inhalation, 8 h WDL, and 72 h WDL + 2BC, respectively.

To examine the relationship between voluntary 2BC EtOH consumption and GABA_A_R gene expression, we used qRT-PCR to semi-quantitatively measure the α4 and the δ subunit mRNA expression levels in the hippocampi of the CIE and the Air group mice. As shown in **Figure 4**, the differences in GABA_A_R α4 and δ subunit mRNA levels between the two groups at the end of the last 2BC drinking session were not statistically significant (*p* = 0.4750 and *p* = 0.2809, respectively).

Furthermore, to better understand the possible contribution of the withdrawal effect, we compared the α4 and the δ subunit transcript levels in the CIE mice at three additional, different time points along the paradigm. We found the highest δ mRNA expression levels at the end of the last EtOH vapor exposure. These levels decreased significantly (–25.5 ± 2.3%, *p* < 0.001) after 8 h of withdrawal from the inhalation chambers; then, a significant increase (+19.2 ± 0.4%, *p* < 0.01) was observed after the first subsequent 2BC drinking session, which was not robust enough to restore pre-withdrawal expression levels (–14.2 ± 0.3%, *p* < 0.01; **Figure 4A**). Conversely, in the case of the α4 subunit, differences in the mRNA expression levels at these three different time points were not statistically significant (**Figure 4B**).

**FIGURE 5:**
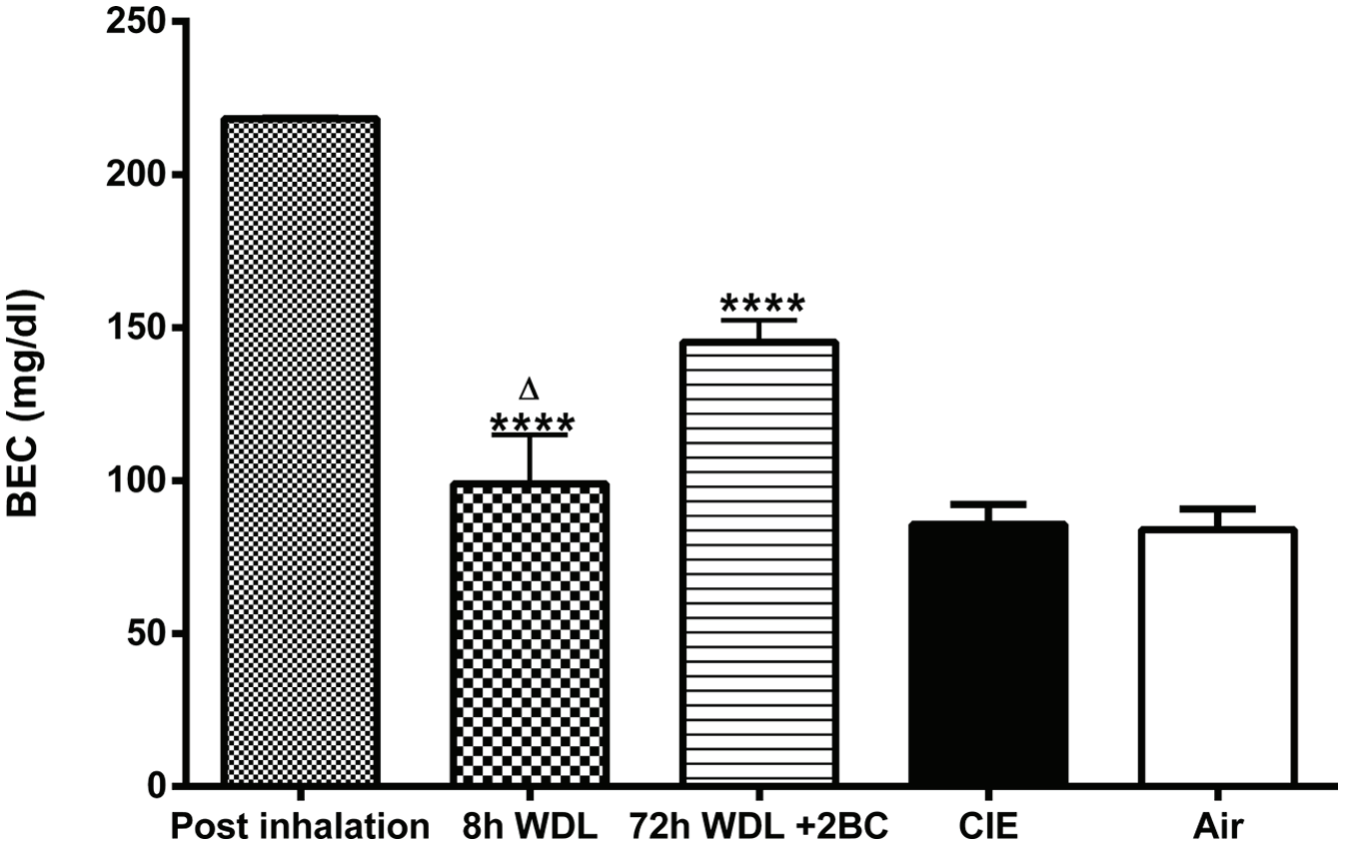
**Effects of repeated cycles of CIE paradigm on plasmatic EtOH levels across different time points.** BECs were measured immediately after samples collection. BECs are expressed in mg/dl. The first three bars refer to the CIE mice immediately after the last forced exposure to EtOH vapor (Post inhalation), 8 h after the last exposure to EtOH vapor (8 h WDL), and right after the first subsequent 2BC drinking session (72 h WDL + 2BC). Statistical differences among BECs at these three different time points are indicated. The two bars on the right refer to BECs in the CIE and the Air mice at the end of the paradigm, immediately after the last 2BC session in the last test week. At this stage, the difference between the average BECs of the two groups was not statistically significant [CIE (*n* = 15) and Air (*n* = 15); *p* = 0.8945; *t*-test: *t* = 0.1337 (30); *R*^2^ = 0.0005955 and *F* test to compare variances *F* = 1.023 (15,15)]. Multiple comparison ANOVA of the first three time points showed significance between treatments with *p* < 0.0001, *F* = 34.60 (2,18), followed by the Bonferroni multiple comparison *post hoc* test, ^∗∗∗∗^*p* < 0.0001 vs. Post inhalation; Δ*p* < 0.05 between 8 h WDL and 72 h WDL + 2BC. The values are means ± SEM of 6, 6, and 9 mice for post inhalation, 8 h WDL, and 72 h WDL + 2BC, respectively.

**FIGURE 6:**
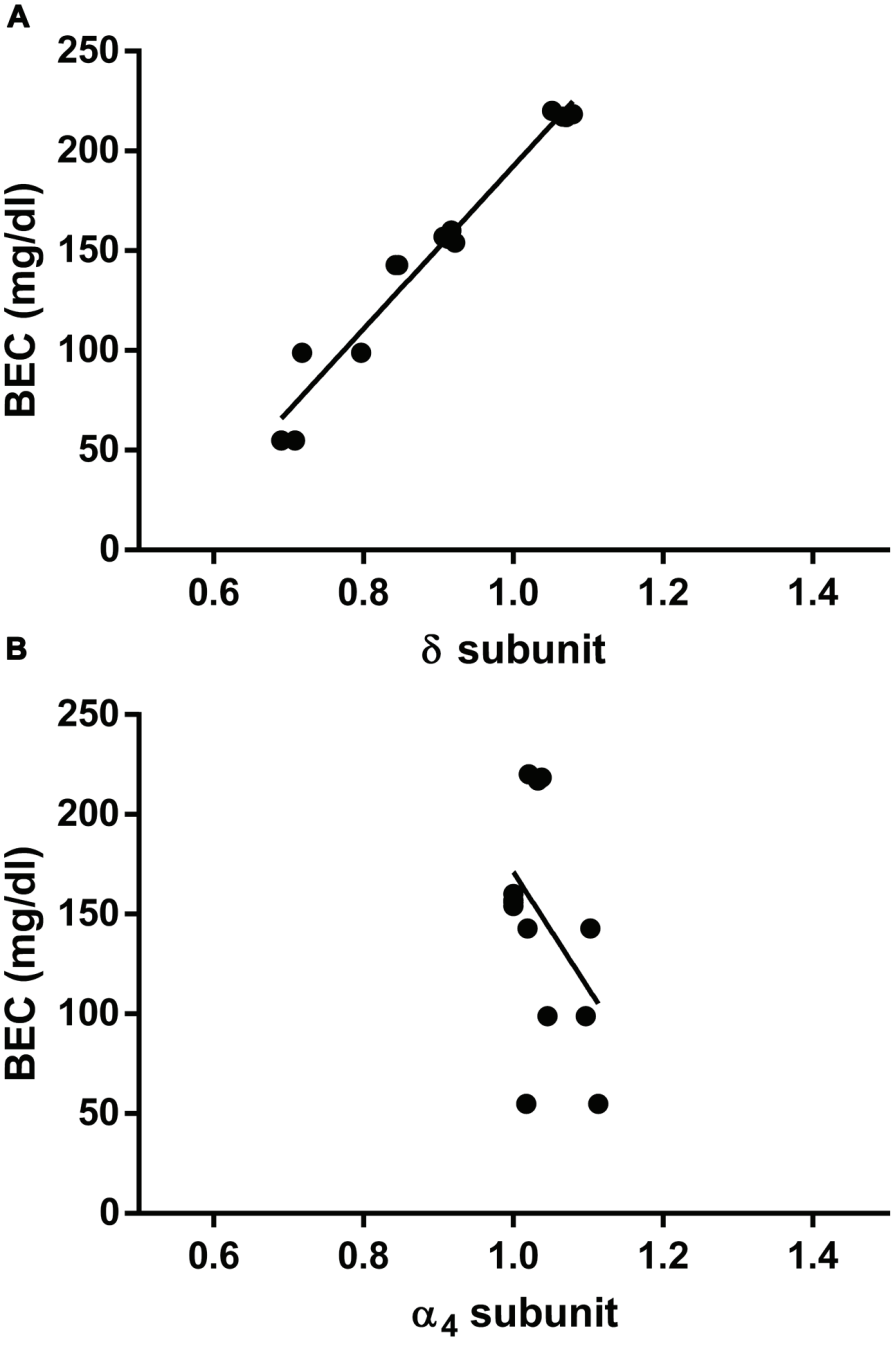
**Correlation between hippocampal GABA_A_R subunit mRNA expression and concomitant plasma EtOH levels measured at different time points in CIE paradigm.** Correlations were calculated for the GABA_A_R δ **(A)** and the α4 **(B)** subunit levels. Data corresponding to the first three time points shown in **Figures 4** and **5** (Post inhalation, 8 h WDL, and 72 h WDL + 2BC) were analyzed. BECs are expressed in mg/dl. GABA_A_R subunit mRNA expression semi-quantitative levels were obtained by qRT-PCR, and gene fold expression levels were normalized with housekeeping mRNA. Pearson’s Product Moment analysis showed a significant correlation for the δ subunit (*R*^2^ = 0.9620; *p* < 0.0001) but not for the α4 (*R*^2^ = 0.1635; *p* = 0.1516). Linear regression analysis showed a slope different from zero for the δ subunit [*p* < 0.0001; *F* = 303.4 (1,12)] but not for the α4 subunit [*p* = 0.1516; *F* = 2.345 (1,12)].

Finally, to evaluate whether the measured fluctuations in the GABA_A_R δ subunit transcript abundance were accompanied by changes in blood EtOH levels, we compared BECs of the CIE mice at the same three time points considered above. Remarkably, we found significant differences among BECs at these three different time points, with higher EtOH levels immediately after inhalation, lower (–55 ± 7.4%, *p* < 0.0001) after 8 h of withdrawal, and intermediate (–33 ± 3.2%, *p* < 0.0001) after the first subsequent 2BC drinking (**Figure 5**). Accordingly, when we correlated these changes in BECs with the mRNA expression levels at the same time points, we found a statistically significant positive correlation with the δ subunit (*R*^2^ = 0.9620; *p* < 0.0001; **Figure 6A**) but no correlation with α4 (**Figure 6B**), suggesting that the high BECs reached during forced inhalation, combined with repeated withdrawal experiences, may induce profound molecular changes which include the specific effects on GABA_A_R gene expression that were not more evident after subsequent voluntary EtOH intake.

## Discussion

A deeper understanding of the molecular adaptation mechanisms occurring after prolonged excessive EtOH consumption, including behavioral, neurochemical, and functional, is essential for developing novel therapeutic approaches to treat alcohol use disorders. Several models of excessive EtOH consumption have been established to explore the progression that leads to dependence, including Chronic Intermittent EtOH exposure, with (Becker and Lopez, 2004; Finn et al., 2007; Griffin et al., 2009a,b; Gorini et al., 2013) or without (Melendez et al., 2012; Maldonado-Devincci et al., 2014; Osterndorff-Kahanek et al., 2015; Staples et al., 2015; Uys et al., 2015) combined 2BC voluntary consumption.

In our study, C57BL/6J mice were initially allowed to self-administer EtOH until the positive reinforcing effects of EtOH were established. Then, the mice were subjected to four cycles of forced exposure to EtOH vapor, followed by EtOH withdrawal, and separated by weekly tests of 2BC voluntary intake. This model is intended to mimic human EtOH consumption, where periods of high intake are followed by abstinence owing to medical aid or treatment, and possibly subsequent relapse.

The escalation of voluntary EtOH drinking previously observed over several successive cycles of CIE exposure (Lopez and Becker, 2005; Lopez et al., 2012) was confirmed in our experiments (**Figure 2A**). While the intensity and duration of EtOH exposure play a major role in contributing to the escalation of drinking in dependent animals (Griffin et al., 2009a), the biological mechanisms driving such escalation remain unclear and all minor variations in the paradigm can potentially influence intake levels.

As reported previously (Becker and Lopez, 2004; Lopez and Becker, 2005), our results show that mice exposed to Chronic Intermittent EtOH vapor inhalation consumed higher volumes of EtOH compared to the controls (**Figures 2A,C**) when subjected to a free-choice paradigm. The increased EtOH consumption is not due to the need to hydrate because the animals were not deprived of water at any time during the experiment. This higher intake level accompanied by a compulsive drinking habit could be explained by an altered physiological state associated with tolerance and dependence. In the CIE animals, such behavior produces cerebral and plasmatic EtOH concentrations similar to those reached during prior forced exposure in inhalation chambers (Griffin et al., 2009b) and was thus likely to re-establish a similar intoxication experience along with the related positive reinforcement properties. Our data show consistently that blood EtOH levels after the first 2BC session following withdrawal were markedly higher compared to those measured at the end of the paradigm (**Figure 5**), although they were lower than those during vapor exposure.

At the end of the 72-h abstinence period following withdrawal from EtOH vapor, the CIE mice exhibited a faster rate of EtOH intake compared to the controls (Griffin et al., 2009b). Thus, as they developed compulsive and anxious behavior leading them to rapidly consume greater amounts of EtOH, they escaped from unpleasant withdrawal symptoms or at least partially cleansed the negative effects of withdrawal (Becker, 2000; Heilig et al., 2010). Indeed, multiple studies suggest that CIE exposure induces a hyperglutamatergic state in the nucleus accumbens (Griffin et al., 2014), where changes in the dendritic spine morphology (Uys et al., 2015) could contribute to the augmented rewarding effects of EtOH observed after self-administration in different rodent models (Roberts et al., 2000; O’Dell et al., 2004; Gilpin et al., 2008; Lopez and Becker, 2014; Vendruscolo and Roberts, 2014). As described in our results, the CIE and the Air mice showed higher EtOH consumption during the first day of the 2BC sessions following the withdrawal periods, with the CIE mice consuming a greater amount of EtOH compared to the Air group (**Figures 2B,C**). Such an effect was not as evident in previous reports (Becker and Lopez, 2004; Finn et al., 2007; Gorini et al., 2013), where CIE mice tended to drink less EtOH during the first 2BC day in comparison with the subsequent days. The fact that the Air and the CIE mice showed a similar pattern of initial consumption suggests that their drinking behavior is driven in part by the consequences of withdrawal, which was longer in the Air group.

Although the amounts of EtOH consumed on the first day of 2BC were higher in the CIE compared to the Air mice (**Figure 2B**), they were also higher than the 7-day average values for CIE mice (**Figure 2C**). When analyzing daily intake during the last 2BC test, we found that despite the substantial gap in EtOH intake in the CIE mice vis-à-vis the Air mice on the first day, the two groups ended up with more comparable EtOH consumption levels (**Figure 3A**) and similar BECs (**Figure 3B**) on the very last day of the paradigm, indicating that repeated cycles of intermittent EtOH vapor exposure did not alter EtOH pharmacokinetics in CIE mice, which corroborates the results of previous studies (Becker, 1999; Lopez et al., 2012).

Collectively, behavioral data suggest that persistent cycles of forced exposure to EtOH followed by withdrawal and voluntary intake are likely associated with gaining relief from withdrawal symptoms, greater rewarding effects, and tolerance, which all promote and maintain excessive dependence-associated consumption. Nevertheless, complex molecular dynamics might contribute to these behavioral responses. Therefore, a major goal of our study was to evaluate the molecular changes underlying the adaptation of the hippocampal GABAergic system to Chronic Intermittent EtOH vapor exposure combined with 2BC voluntary intake.

We have previously shown that chronic EtOH exposure can affect the GABA_A_R gene expression of hippocampal neurons in culture (Follesa et al., 2005) and in mice (Sanna et al., 2011). Here, we focused on the α4 and the δ subunit mRNA expressions, which are sensitive to acute EtOH modulation (Sundstrom-Poromaa et al., 2002; Wallner et al., 2003; Wei et al., 2004), following a CIE exposure paradigm. In agreement with consumption data, we did not find significant changes in the α4 and the δ subunit transcript levels between groups at the end of the paradigm (**Figure 4**). This might suggest that putative perturbations in the α4 and the δ levels could have been transient in the CIE mice and related to the differences in EtOH consumption between the groups, which were less pronounced at that stage.

Surprisingly, we did not find significant changes in the α4 subunit mRNA expression when we compared normalized transcript levels at three additional time points across the paradigm (**Figure 4B**), although it had been shown previously that this GABA_A_R subunit undergoes rapid fluctuations during EtOH withdrawal in cell cultures (Follesa et al., 2003, 2005) and that its overexpression is associated with long-term dependence in alcoholics (Jin et al., 2012), greater anxiety in animal models (Smith et al., 1998b; Cagetti et al., 2003), and reduced neurosteroid levels during social isolation (Serra et al., 2006) or after delivery (Sanna et al., 2009). Our group has previously reported that social isolation induced increased hippocampal α4 and δ subunit gene expression in C57BL/6J mice and that stress-induced EtOH 2BC self-administration can prevent changes in α4 but not in the δ subunit (Sanna et al., 2011). Conversely, in the present study it is possible that we might not have been able to detect similar variations in α4 gene expression owing to potentially altered responses to stress in the CIE dependent animals (Kumar et al., 2009; Heilig et al., 2010; Mody and Maguire, 2011), concurrent modifications of other GABA_A_R subunits that can be assembled with δ (Glykys et al., 2007; Suryanarayanan et al., 2011), transport of α4-containing GABA_A_Rs from extrasynaptic to synaptic locations following EtOH exposure (Liang et al., 2006), and long-lasting changes in hippocampal structural plasticity (i.e., dendritic arborization) induced by CIE exposure (Staples et al., 2015).

Remarkably, when we compared hippocampal δ subunit mRNA expression at different time points in the CIE mice, we found an initial upregulation after EtOH vapor exposure, followed by a sudden downregulation 8 h after their removal from the inhalation chambers (**Figure 4A**). This decrease suggests a molecular adaptation in the hippocampal GABAergic system to partially recover the neuronal excitability compromised by EtOH intoxication, eventually leading to increased excitability with higher avidity for EtOH, which is typical of withdrawal conditions. After the subsequent first day of 2BC voluntary drinking, a significant net upregulation in δ mRNA levels partially recovered the subunit expression, but failed to restore pre-withdrawal transcript levels (**Figure 4A**). Our group has previously reported δ subunit upregulation in the hippocampi of C57BL/6J mice subjected to voluntary EtOH consumption (Sanna et al., 2011). Thus, δ upregulation observed after 2BC EtOH consumption following abstinence could re-establish neuronal excitability to partially overcome the opposite regulation observed during withdrawal.

Moreover, when we compared BECs in the CIE mice at the same three time points, we found significant differences, which were parallel to the observed fluctuations in GABA_A_R δ subunit transcript abundance (**Figure 5**). This supports the view that reduced blood and, thus, brain EtOH concentrations during EtOH withdrawal induce molecular neuroadaptations in hippocampal GABAergic transmission. These changes might, in turn, possibly contribute to an increase in the intake of and avidity for EtOH when it is presented again for voluntary drinking. Additionally, fluctuations in the δ subunit might partly depend on exposure/consumption because they were directly correlated to the resulting BECs, at least in the three stages examined (**Figure 6A**), resembling the molecular and functional changes observed in cultured hippocampal neurons by varying EtOH concentration (Follesa et al., 2005). Thus, a general increase in δ-containing hippocampal GABA_A_Rs might represent a physiological response to high EtOH concentrations during forced exposure to EtOH vapor, as well as after self-administration. However, the interpretation of its biological significance is complicated by the fact that the technique used did not allow for discrimination among the different hippocampal subregions, cellular subpopulations, and GABA_A_Rs localization.

Nevertheless, the reported changes in the expression levels of δ-containing GABA_A_Rs, associated with corresponding shifts in the hippocampal GABAergic tonic current (Sanna et al., 2011), involve alterations in the physiological response to stress and anxiety (Sarkar et al., 2011; Whissell et al., 2015), which ultimately change the sensitivity of mice, sustaining addictive behavior (Heilig et al., 2010).

In the present study, the transient withdrawal-induced decrease in δ subunit mRNA levels, observed 8 h after the fourth EtOH vapor cycle, might be accompanied by an internalization of δ-containing GABA_A_Rs and a complementary increase of γ-containing receptors, which are less sensitive to EtOH and are able to mediate synaptic currents. These transient adaptations might recur with each 72-h abstinence cycle and entail more severe, long-lasting changes in hippocampal cellular morphology (Staples et al., 2015) and in the plasticity of reward neurocircuitry (Uys et al., 2015), which could contribute to relapse vulnerability and escalate intake levels over time. Therefore, it would be interesting to monitor BECs and complementary molecular changes in additional subunits during each withdrawal cycle, test direct causality for such complex drinking-related regulation of GABA_A_R subtype expression, and include earlier time points to better cover the temporal evolution of the observed changes.

Our study provides new evidence pertaining to the dynamic regulation of the GABA_A_R δ subunit during alcohol dependence, contributes to the understanding of its role in other addictive and comorbid conditions, and suggests possible behavioral implications, which may be important for individual responsiveness to therapeutic drugs.

## Conflict of Interest Statement

The authors declare that the research was conducted in the absence of any commercial or financial relationships that could be construed as a potential conflict of interest.
